# Aerobic exercise protects against pressure overload-induced cardiac dysfunction and hypertrophy via β3-AR-nNOS-NO activation

**DOI:** 10.1371/journal.pone.0179648

**Published:** 2017-06-16

**Authors:** Bin Wang, Ming Xu, Wenju Li, Xiaoli Li, Qiangsun Zheng, Xiaolin Niu

**Affiliations:** 1 Department of Cardiology, Tangdu Hospital, Fourth Military Medical University, Xi’an, China; 2 Department of Physiology, School of Basic Medicine, Shanghai University of Traditional Chinese Medicine, Shanghai, China; 3 Department of Cardiology, the Second Affiliated Hospital of Xi’an Jiaotong University, Xi’an, China; Emory University, UNITED STATES

## Abstract

Aerobic exercise confers sustainable protection against cardiac hypertrophy and heart failure (HF). Nitric oxide synthase (NOS) and nitric oxide (NO) are known to play an important role in exercise-mediated cardioprotection, but the mechanism of NOS/NO stimulation during exercise remains unclear. The aim of this study is to determine the role of β3-adrenergic receptors (β3-ARs), NOS activation, and NO metabolites (nitrite and nitrosothiols) in the sustained cardioprotective effects of aerobic exercise. An HF model was constructed by transverse aortic constriction (TAC). Animals were treated with either moderate aerobic exercise by swimming for 9 weeks and/or the β3-AR-specific inhibitor SR59230A at 0.1 mg/kg/hour one day after TAC operation. Myocardial fibrosis, myocyte size, plasma catecholamine (CA) level, cardiac function and geometry were assessed using Masson’s trichrome staining, FITC-labeled wheat germ agglutinin staining, enzyme-linked immuno sorbent assay (ELISA) and echocardiography, respectively. Western blot analysis was performed to elucidate the expression of target proteins. The concentration of myocardial NO production was evaluated using the nitrate reductase method. Myocardial oxidative stress was assessed by detecting the concentration of myocardial super oxidative dismutase (SOD), malonyldialdehyde (MDA), and reactive oxygen species (ROS). Aerobic exercise training improved dilated left ventricular function and partially attenuated the degree of cardiac hypertrophy and fibrosis in TAC mice. Moreover, the increased expression of β3-AR, activation of neuronal NOS (nNOS), and production of NO were detected after aerobic exercise training in TAC mice. However, selective inhibition of β3-AR by SR59230A abolished the upregulation and activation of nNOS induced NO production. Furthermore, aerobic exercise training decreased the myocardial ROS and MDA contents and increased myocardial levels of SOD; both effects were partially attenuated by SR59230A. Our study suggested that aerobic exercise training could improve cardiac systolic function and alleviate LV chamber dilation, cardiac fibrosis and hypertrophy in HF mice. The mechanism responsible for the protective effects of aerobic exercise is associated with the activation of the β3-AR-nNOS-NO pathway.

## Introduction

Heart failure (HF) is a major cause of death worldwide. It is estimated that 23 million patients are affected by HF worldwide [1]. Although progress has been made in the diagnosis and treatment of HF, the 5-year mortality rate of HF remains as high as 45%–60% [2]. Therefore, identifying an effective therapeutic target to improve cardiovascular function is critical.

Moderate aerobic exercise training has many beneficial effects on the cardiovascular system. Exercise can decrease the incidence of numerous cardiovascular diseases and improve ventricular mechanical performance and function; for example, exercise can lead to improved aerobic fitness (VO2max), enhanced cardiac contraction, and accelerated relaxation [3]. Meanwhile, aerobic exercise training in patients with stable HF can also relieve patients’ symptoms, improve their exercise capacity and quality of life, and reduce disability, hospitalization, and mortality [4]. Previous studies have suggested that nitric oxide synthase (NOS) and nitric oxide (NO) play an important role in exercise-mediated cardioprotection [3]. According to previous studies, levels of the NO metabolites nitrite and nitrosothiols were increased during exercise in both rodents and humans plasma [5,6], and heart NOS expression was elevated during exercise in a nitric oxide-deficient hypertension model [7]. However, the mechanism of NOS/NO stimulation during exercise remains unclear.

β3-adrenergic receptors (β3-ARs) are crucial regulators of cardiovascular function in response to stress; these receptors appear to exert a negative inotropic effect and are activated by catecholamines at higher concentrations than are β1/β2-ARs [8,9]. The sympathetic nervous system (SNS) is overactive in HF, leading to worsened heart function with disease progression [10]. β1/β2-ARs are down-regulated or desensitized during HF[11]. However, β3-ARs are up-regulated in failing hearts [12]. Accumulating evidence has demonstrated that β3-ARs represent a potential target for the treatment of cardiovascular diseases, including hypertension, acute myocardial infarction (MI), and HF [13]. Napp et al. [14] suggested that the cardioprotective effects of β3-ARs are associated with NO release via NOS activation. Our previous studies have suggested that pressure overload in β3-AR knockout mice resulted in increased NOS uncoupling, leading to increased LV dilation and worsened heart function [8]. We also showed that a specific β3-AR agonist significantly attenuated myocardial hypertrophy and preserved heart function in mice with HF via NOS/NO activation [15].

Catecholamine stimulates β3-AR, and the cardioprotective effects of β3-AR agonists in mice with HF are associated with NO and NOS [15]. Moreover, NO metabolites and catecholamine levels increase during exercise[5,6,16]. We speculate that the β3-AR-NOS-NO pathway participates in the cardio protective effects of exercise training against HF. Thus, we designed the present study to explore the protective effects of exercise training in an established in vivo mouse model of transverse aortic constriction (TAC) and to investigate the role of β3-ARs in mediating the cardioprotective effects of exercise.

## Methods

### Animals

The present study consisted of two parts. In the first part, 60 adult male C57BL6/J mice (weighing 18 to 21 g, 8 weeks old) were randomly divided into the following groups, with n = 15 each: (1) sham-operated control group (SHAM); (2) sham-operated control + Exercise training group (SHAM+E); (3) TAC group (TAC); and (4) TAC + Exercise training group (TAC+E). The HF model was constructed using TAC as previously described[15]. The mice in the TAC and TAC+E groups underwent TAC to induce cardiac hypertrophy and HF via pressure overload. The SHAM and SHAM+E groups underwent the same surgical procedures, except that the suture under the transverse aorta was not tied. In the second part, 45 adult male C57BL6/J mice (weighing 18 to 21 g, 8 weeks old) were obtained and arbitrarily divided into the following groups, with n = 15 each: (1) TAC group (TAC); (2) TAC + Exercise training group (TAC+E); and (3) TAC + Exercise + SR59230A group (TAC+E+SR). The mice underwent TAC to induce cardiac hypertrophy and HF. The mice in TAC+E+SR were administered the specific β3-AR antagonist SR59230A at 0.1 mg/kg/hour via osmotic mini-pumps (Alzet Inc, Cupertino, CA) one day after the TAC operation and continued until the end of the study. The mice in TAC and TAC+E group were administered PBS (the vehicle of SR59230A) via osmotic mini-pumps. All the animals were obtained from the animal center of the Fourth Military Medical University, and housed in a temperature-controlled animal facility with a 12-hour light/dark cycle, and fed a normal chow diet provided ad libitum. Mice were sacrificed one day after the last swimming training in the aerobic exercise protocol. Hearts were separated and immediately stored at -80°C or fixed in 4% paraformaldehyde. The plasma samples were taken and stored at -80°C. All experimental procedures were approved by the Fourth Military Medical University Committee on Animal Care and were performed in adherence with the National Institutes of Health Guidelines on the Use of Laboratory Animals. Mice were euthanized by cervical dislocation under deep anesthesia with isoflurane (4%), and all efforts were made to minimize suffering.

### Exercise protocol

Mice in the SHAM+E, TAC+E and TAC+E+SR groups were trained via swimming one week after TAC or sham operation. Training was performed 5 days per week in a bucket, following a progressive 9-week program. The water temperature was controlled at 32–33°C. We observed the whole process of swimming, no mouse was just floating during swimming under the impact of the other mice. The training schedule is described in Table 1.

**Table 1:** Description of the training protocol.

	week 1	weeks 2–3	weeks 4–5	weeks 6–7	weeks 8–9
Exercise duration per day (min)	20	30	40	50	60

### Echocardiographic measurements of cardiac function and geometry

In vivo cardiac geometry and function were assessed using transthoracic echocardiography at baseline, 1 week, 3 weeks, 5 weeks and 9 weeks until the mice were sacrificed at 10 weeks after TAC. The body weights were measured, and the mice were then placed in a supine position and anesthetized (2% isoflurane and oxygen). Both two-dimensional and M-mode images were recorded using a 30-MHz transducer on a Vevo 2100 ultrasound system (Visual Sonics, CA). The left ventricular mass (LVM), left ventricular end-diastolic diameter (LVEDd), left ventricular end-systolic diameter (LVESd), left ventricular ejection fraction (LVEF), fractional shortening (FS), interventricular septal thickness (IVS) and left ventricular posterior wall thickness (LVPW) were measured. Echocardiography was evaluated in a blinded manner.

### Western blotting

Western blotting was performed to analyze protein expression; the protocol used was described previously [17]. Fresh-frozen LV tissue was homogenized in cell lysis buffer (containing Roche phosphatase inhibitor cocktail and proteinase inhibitor PMSF), and total protein was measured using a Bradford protein assay. Equal amounts of protein sample (50 μg) were separated by electrophoresis on 12% SDS-PAGE gels in a Tris/ HCl buffer system, sequentially electrophoretically transferred to polyvinylidene fluoride (PVDF) membranes. After blocking with 5% nonfat milk in Tris-buffered saline containing 0.05% Tween-20 (TBST), PVDF membranes were subjected to immunoblotting with appropriate primary antibodies at 4°C over night, followed by incubation with appropriate horseradish peroxidase conjugated secondary antibodies at 37°C for 60 min. Blots bands were detected via enhanced chemiluminescence (Millipore) and visualized with the Molecular Imager ChemiDoc XRS+ system (BIO-RAD, USA). Densitometric analyses were then performed using Lab Image software. Three replicates were performed for each time point.

The following primary antibodies were used: eNOS (1:400, Santa Cruz Biotechnology), Phospho-eNOS Ser1177 (p-eNOS ^Ser1177^) (1:1000, Abcam), Phospho-eNOS Ser114 (p-eNOS ^Ser114^) (1:1000, Millipore Corporation), nNOS (1:1000, Abcam), Phospho-nNOS Ser847 (p-nNOS ^Ser847^) (1:600, Abcam), Phospho-nNOS Ser1412 (p-nNOS ^Ser1412^) (1:600, Abcam), β1-adrenergic receptor (1:400, Santa Cruz Biotechnology), β2-adrenergic receptor (1: 400, Santa CruzBiotechnology), β3-adrenergic receptor (1:400, Santa Cruz Biotechnology), GAPDH (1:5000, Abcam).

### Histological evaluation of myocyte size and myocardial fibrosis

Mice were sacrificed 10 weeks after the TAC operation, and samples were prepared for histological assays. The hearts were harvested, fixed in 4% paraformaldehyde, and sectioned into three equal divisions perpendicular to the LV long axis. The mid-ventricular segment was embedded in paraffin, and sections were prepared at 4 μm thickness. The anterolateral sections were evaluated. FITC-labeled wheat germ agglutinin (Sigma) staining was performed to detect cardiomyocyte cross sectional area[18]. The cardiac myocyte membranes were observed by fluorescence microscopy. Morphometric analysis was performed with Image-Pro Plus software. Only cells with well-defined cell membranes were selected. The outline of 100 myocytes were traced in each group. Masson’s trichrome staining was performed to detect fibrosis in cardiac muscle. Ten random microscopic fields from each tissue section (two animals per group) were digitally captured under the fixed microscope illumination settings. Image-Pro Plus software was used to calculate the scar extent.

### Measurement of plasma catecholamine secretion

Plasma epinephrine (Epi) and norepinephrine (NEpi) levels were determined by enzyme-linked immunosorbent assay(ELISA), performed on mice plasma samples using the mouse catecholamine ELISA Kit (JiangLai Bioengineering Corporation, China), as described previously[19]. The samples were taken when mice were sacrificed (Basal). The samples were anticoagulated with heparin, then centrifuged at 1000 g for 20 min. The supernatant were used for catecholamine detecting. The absorbance was measured at 450 nm, and the catecholamine levels (pg/ml) were determined using a standard curve. In order to determine whether the plasma catecholamine levels would increse during aerobic exercise, we measured the plasma catecholamine levels at basal (no exercise for 1 day) and right after aerobic exercise (swimming for 20 mins) two weeks after the TAC operation. Mice were anesthetized with isoflurane, and the blood samples were collected from inferior vena cava.

### Assessment of cardiac NO production

Cardiac NO production was determined by evaluation of its oxidation products (nitrate and nitrite) using the nitrate reductase method described by Miranda et al [20]. The nitrate was reduced to nitrite by nitrate reductase, and the nitrite was measured by the Griess reaction. Fresh-frozen LV myocardium was converted to homogenates in a homogenizer filled with precooled normal saline (NS). The homogenates were centrifuged at 4°C for 15 mins with a speed of 2000 r/min. The supernatant were mixed with the reagents supplied in an NO Assay Kit (Nanjing Jiancheng Bioengineering Corporation, China, A012) and incubated at 37°C for 60 min. The absorbance was measured spectrophotometrically at 530 nm. All operations were according to the manufacturer’s instructions. The total NO content (μmol/g prot) was determined using a standard curve. Each preparation was tested in triplicate.

### Measurement of reactive oxygen species (ROS) production

Immunofluorescence was used to measure ROS levels in the myocardium. Mice were sacrificed 10 weeks after the TAC operation. Fresh-frozen myocardium was serially sectioned at 4 μm thickness and incubated with 2,7-dichlorofluorescin diacetate (DCFH-DA) (20 μM) (ROS assay kit, Nanjing Jiancheng Bioengineering Corporation, China, E004) at 37°C for 60 min in the dark. Five random microscopic fields from each tissue section (two animals per group) were viewed under an Olympus IX71 fluorescence microscope (Tokyo, Japan). The fluorescence intensity of the sections stained for ROS were calculated using Image-Pro Plus software.

### Tissue malondialdehyde (MDA) analysis

MDA levels in LV myocardial tissue were determined using the thiobarbituric acid (TBA) method. Fresh-frozen LV myocardium was converted to homogenates in a homogenizer filled with precooled normal saline (NS). The homogenates were centrifuged at 4°C for 15 mins with a speed of 2000 r/min. The supernatant were mixed with the reagents supplied in an MDA Assay Kit (Nanjing Jiancheng Bioengineering Corporation, China, A003-2) and incubated at 95°C for 40 min. After cooling at room temperature, the mixture was centrifuged at 4000 g for 10 min. The absorbance of the supernatant was measured at 530 nm. All operations were according to the manufacturer’s instructions. The MDA concentrations were expressed as nmol/mg prot.

### Measurement of superoxide dismutase (SOD) activity

The total SOD activity in LV myocardium was determined using the hydroxylamine method. Fresh-frozen LV myocardium was converted to homogenates in a homogenizer filled with precooled normal saline (NS). The homogenates were centrifuged at 4°C for 15 mins with a speed of 2000 r/min. The supernatant were mixed with the reagents supplied in a SOD Assay Kit (Nanjing Jiancheng Bioengineering Corporation, China, A001-3). The mixture was incubated at room temperature for 10 min, and the absorbance of the compound was then measured at 550 nm. All operations were according to the manufacturer’s instructions. Each preparation was tested in triplicate. SOD activity was expressed as U/mg prot.

### Statistical analysis

All data are presented as the mean ±standard error of the mean. Statistical analysis was performed using GraphPad Prism 5.0 (San Diego, CA, USA). Statistical comparisons of serially measured cardiac parameters were performed using a repeated-measures analysis of variance (ANOVA) with a post hoc test for comparisons between groups. Statistical comparisons of other data were performed using one-way ANOVA with a post hoc test. All P values<0.05 were considered statistically significant.

## Results

### Aerobic exercise prevents the deterioration of cardiac function

**Fig 1:**
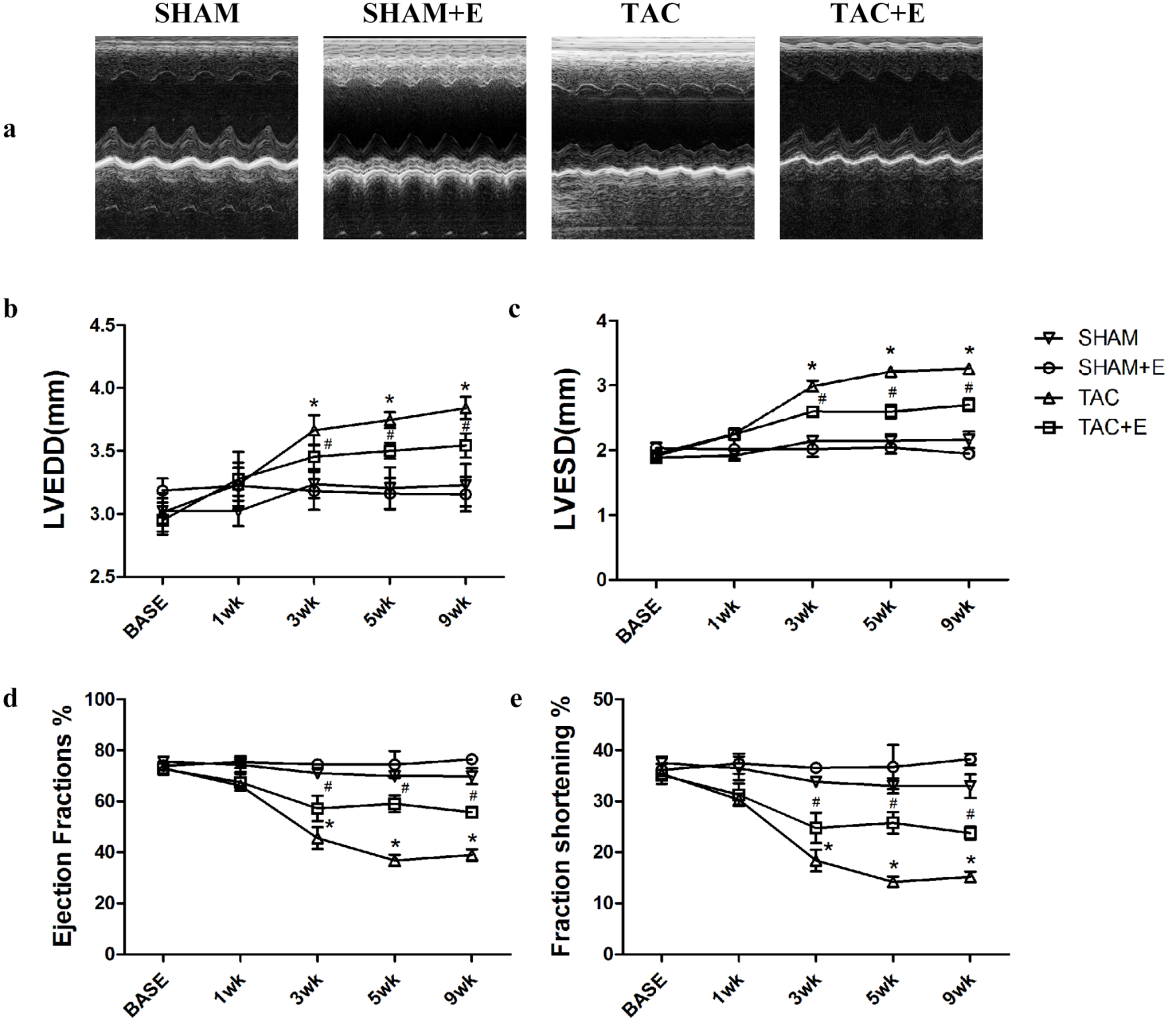
Effects of aerobic exercise on LV dilation and LV systolic function after TAC. (**a**) Representative M-mode echocardiographic images were taken at the level of the papillary muscle, where left ventricular diameters can be measured. Quantification of the left ventricular end diastolic diameter (LVEDd) (**b**), left ventricular end systolic diameter (LVESd) (**c**), left ventricular ejection fraction (EF) (**d**) and fractional shortening (FS) (**e**) 10 weeks after TAC. (**b**) (**c**) (**d**) (**e**) (n = 12 per group. *P<0.05 vs. SHAM. ^#^P<0.05 vs. SHAM and TAC).

Echocardiograms were used to evaluate heart function in all groups. Representative M-mode echocardiography illustrated that mice in TAC group developed decreased systolic function and increased LV dilation after TAC. The LVESd and LVEDd in the TAC group were both higher (Fig 1b and 1c), while the EF% and FS% were lower, than those in the SHAM group (Fig 1d and 1e). However, three weeks of exercise training partly prevented LV dilation and preserved cardiac systolic function. Compared with the TAC group, the TAC+E group displayed decreased LVEDd (3.45±0.10 mm, P<0.05, Fig 1b) and LVESd (2.60±0.08 mm, P<0.05, Fig 1c) and increased EF% (57.25±4.99%, P<0.05, Fig 1d) and FS% (24.76±2.97%, P<0.05, Fig 1e). This exercise-induced protection continued through 9 weeks of training. Additionally, echocardiographic analysis revealed that the baseline parameters were similar in all groups.

### Aerobic exercise reduced myocyte hypertrophy and fibrosis after TAC

**Fig 2:**
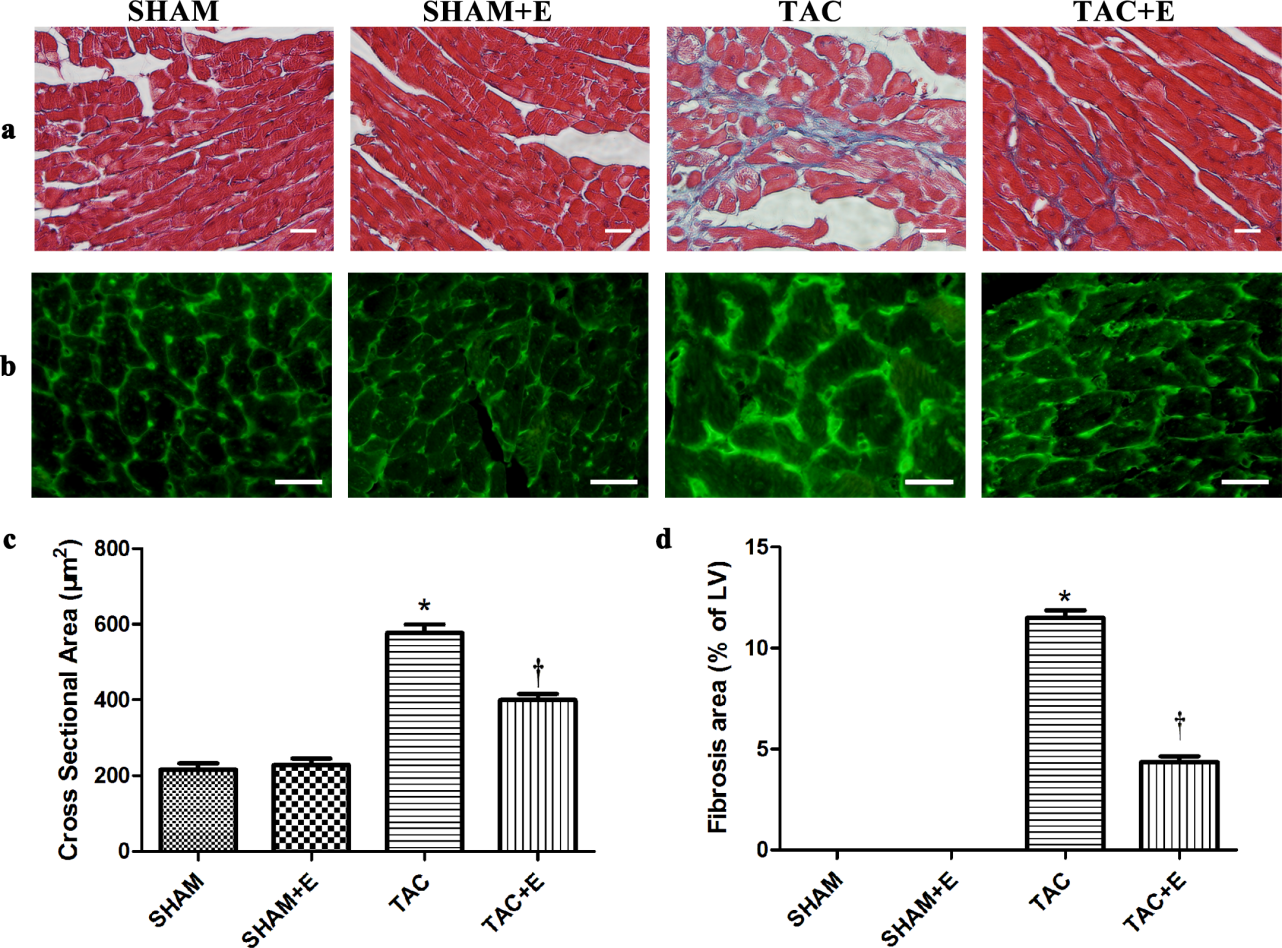
Effects of aerobic exercise on cardiomyocyte cross sectional area and fibrosis induced by TAC. (**a**) Representative Masson’s trichrome staining revealed left ventricular fibrosis 9 weeks after exercise training. Red indicates viable myocardium; blue indicates fibrosis. Scale bar represents 20 μm. (**b**) Representative WGA staining revealed cardiomyocyte cross sectional area. Green fluorescence delineate cardiomyocyte membranes (**c**) Quantitative analysis of cardiomyocyte cross sectional area (n = 100 per group. *P<0.05 vs. SHAM and TAC+E. ^†^P<0.05 vs. SHAM). (**d**) Quantitative analysis of the fibrotic area (n = 20 per group.*P<0.05 vs. SHAM and TAC+E. ^†^P<0.05 vs. SHAM).

FITC-labeled wheat germ agglutinin (WGA) staining and Masson’s trichrome staining were performed to reveal the effects of aerobic exercise on the extent of myocyte hypertrophy and fibrosis after TAC (Fig 2). The cardiomyocyte cross sectional area was significantly increased in TAC mice than SHAM mice(576.79±103.27 μm^2^ vs. 216.41±72.66 μm^2^ in the SHAM group, P<0.05, Fig 2c). Nine weeks of swimming training lessened the cardiomyocyte cross sectional area after TAC(399.93±72.52 μm^2^ vs. 576.79±103.27 μm^2^ in the TAC group, P<0.05, Fig 2c). Severe fibrosis was observed in the hearts of mice in the TAC group (11.51±1.59%, Fig 2d). Compared with mice in the TAC group, mice in the TAC+E group displayed a 65% reduction in fibrosis (4.37±1.22%, P<0.05, Fig 2d).

### Aerobic exercise reduced cardiac hypertrophy after TAC

**Fig 3:**
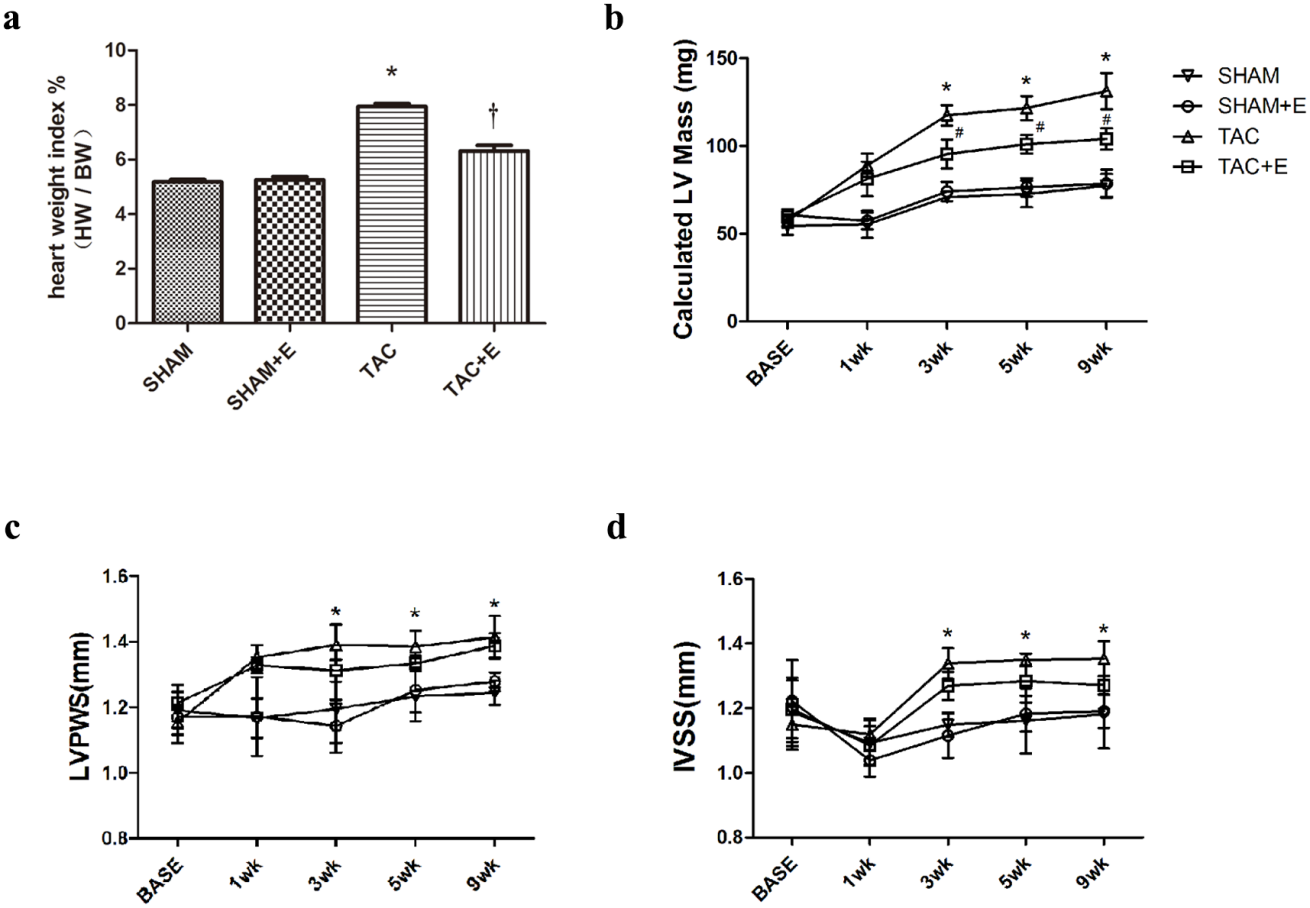
Effect of aerobic exercise on cardiac hypertrophy after TAC. (**a**) Representative heart weight to body weight ratio 10 weeks after TAC. (n = 12 per group. *P<0.05 vs. SHAM and TAC+E. †P<0.05 vs. SHAM). Quantification of calculated left ventricular mass (LVM) (**b**), left ventricular end systolic posterior wall (LVPWS) (**c**), and interventricular septum end systolic thickness (IVSS) (**d**) 10 weeks after TAC. (**b**) (**c**) (**d**) (n = 12 per group. *P<0.05 vs. SHAM. ^#^P<0.05 vs. TAC and SHAM).

Cardiac hypertrophy was increased after TAC (Fig 3). Body weight did not differ significantly among the groups at baseline, 1 w, 3 w, 5 w and 9 w (S1 Fig). The heart weight to body weight ratio in the TAC group was 53% higher than that in the SHAM group (P<0.05, Fig 3a). These data were consistent with the LVM (117.52±5.81 mg in the TAC group vs. 70.90±2.09 mg in the SHAM group, 3 weeks, P<0.05, Fig 3b), LVPW (1.39±0.06 mm in the TAC group vs. 1.20±0.10 mm in the SHAM group, 3 weeks, P<0.05, Fig 3c) and IVS (1.34±0.05 mm in the TAC group vs. 1.15±0.04 mm in the SHAM group, 3 weeks, P<0.05, Fig 3d) calculated using echocardiography. Exercise training reduced the heart weight to body weight ratio by 21% after TAC (P<0.05, Fig 3a). The LVM was also reduced in the TAC+E group compared with the TAC group (P<0.05, Fig 3b). IVS and LVPW did not significantly differ between the TAC and TAC+E groups (Fig 3c and 3d). However, exercise training could not completely prevent cardiac hypertrophy. The heart weight index and LVM were both greater in the TAC+E group than in the SHAM group (P<0.05, Fig 3a and 3b).

### Effects of aerobic exercise on catecholamine levels after TAC

**Fig 4:**
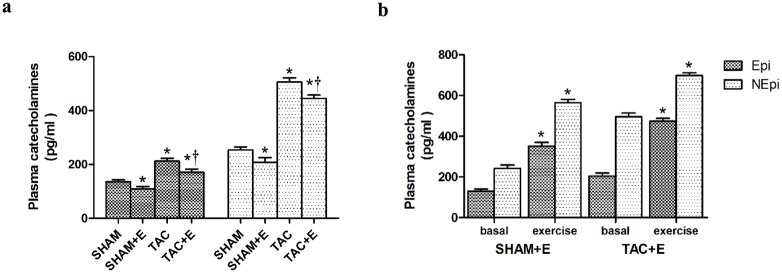
Effect of aerobic exercise on catecholamine levels after TAC. The plasma catecholamine levels determined by enzyme-linked immunosorbent assay (ELISA). (**a**) Quantitative of the plasma catecholamine levels at basal. (n = 5 per group. *P<0.05 vs. SHAM. ^†^P <0.05 vs. TAC) (**b**) Quantification analysis of the plasma catecholamine levels right after exercise. (n = 5 per group. *P<0.05 vs. basal).

As shown in Fig 4a, basal plasma catecholamine levels were increased after TAC (P<0.05 vs. SHAM). 9 weeks aerobic exercise resulted in a reduction of basal plasma catecholamine levels compared with TAC group (P<0.05, Fig 4a). However, the plasma levels of both NEpi and Epi were significantly increased right after exercise (P<0.05, Fig 4b).

### Aerobic exercise increased cardiac β3-AR expression after TAC

**Fig 5:**
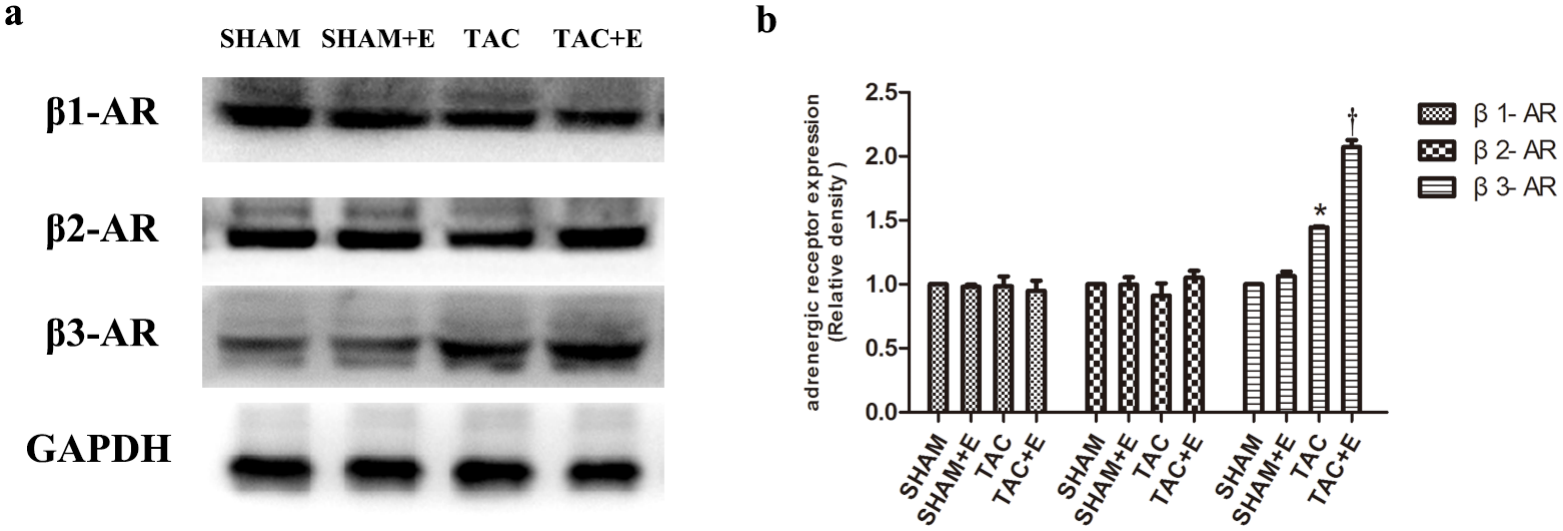
Effects of aerobic exercise on the expression of β-AR subtypes after TAC. (**a**) Representative immunoblots of β1-AR, β2-AR and β3-AR in the SHAM, SHAM+E, TAC and TAC+E groups. (**b**) Semiquantitative analysis of β1-AR, β2-AR and β3-AR expression (n = 6 per group. *P<0.05 vs. SHAM and TAC+E. ^†^P<0.05 vs. SHAM).

Western blotting was performed to investigate cardiac β1/β2-AR and β3-AR protein expression. As shown by representative blotting results and semiquantitative analyses, β3-AR expression was increased in the TAC group compared with the SHAM group (Fig 5b). Moreover, cardiac β3-AR expression was further increased in the TAC+E group (P<0.05 vs. TAC, Fig 5b). Neither β1-AR nor β2-AR expression was changed in any group.

### Aerobic exercise increased cardiac NO production and decreased oxidative stress after TAC

**Fig 6:**
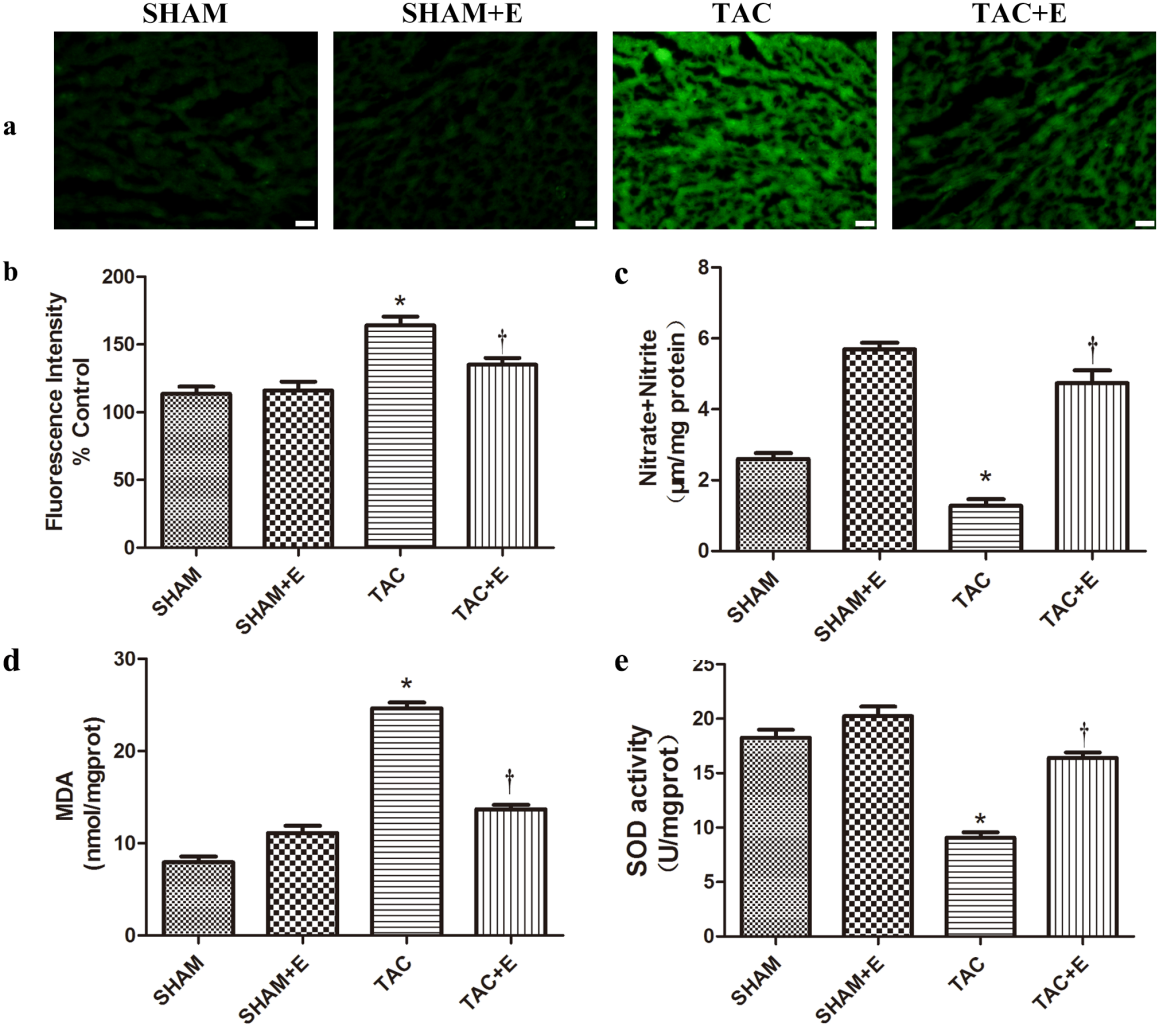
Aerobic exercise increases cardiac NO production and decreases oxidative stress after TAC. (**a**) Representative images of heart sections fluorescently stained for ROS from the SHAM, SHAM+E, TAC and TAC+E groups. Green fluorescence indicates ROS production. Scale bar represents 50 μm. (**b**) Quantitative analysis of the ROS levels (n = 10 per group. *P<0.05 vs. SHAM and TAC+E. ^†^P<0.05 vs. SHAM). (**c**) Quantitative analysis of NO production assessed using the nitrate reductase method 10 weeks after TAC. (**d**) Quantitative analysis of MDA levels assessed using the TBA method. (**e**) Quantitative analysis of SOD levels assessed using the hydroxylamine method. (**c**) (**d**) (**e**) (n = 8 per group. *P<0.05 vs. SHAM and TAC+E. ^†^P<0.05 vs. SHAM).

We tested NO production by summing the concentrations of the NO metabolites (nitrate and nitrite). We also detected ROS production and MDA and SOD levels in the myocardium. As shown in Fig 6, ROS production was increased by 45%, and total nitrate and nitrite concentrations were decreased by 50% in the hearts of TAC mice compared with those of SHAM mice (P<0.05, Fig 6b and 6c). Aerobic exercise training significantly increased the NO metabolite concentration (4.73±0.63 μm/mg prot in the TAC+E group vs. 1.28±0.32 μm/mg prot in the TAC group, P<0.05, Fig 6c) and inhibited ROS generation (135±8.72% in the TAC+E group vs. 164±11.53% in the TAC group, P<0.05, Fig 6b). Levels of MDA and SOD, which indicate free radical metabolism and oxidative stress, were increased by 3.5-fold and decreased by 50%, respectively, in the TAC group compared with the SHAM group (Fig 6d and 6e). Aerobic exercise training can partly prevent the increase in MDA and the decrease in SOD.

### Aerobic exercise modulated nNOS expression and activation

**Fig 7:**
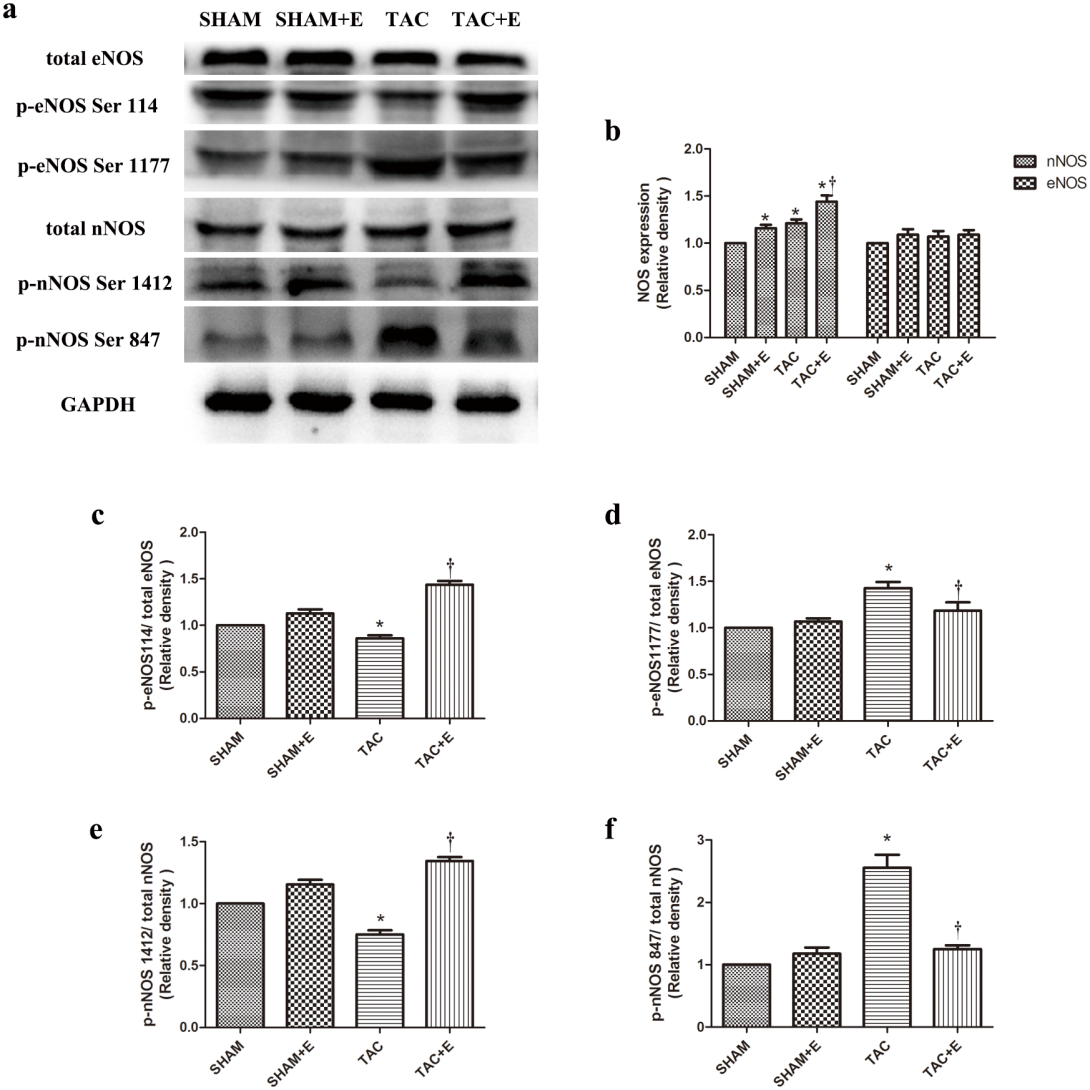
Effects of aerobic exercise on the expression and phosphorylation status of eNOS and nNOS after TAC. (**a**) Representative immunoblots of p-eNOS (Ser1177/Ser114), total eNOS, p-nNOS (Ser1412/Ser847) and total nNOS in the SHAM, SHAM+E, TAC and TAC+E groups. Semiquantitative analysis of eNOS and nNOS expression (**b**) (n = 6 per group. *P<0.05 vs. SHAM. ^†^P<0.05 vs. TAC). Semiquantitative analysis of p-eNOS Ser114 (**c**), p-eNOS Ser1177 (**d**), p-nNOS Ser1412 (**e**), and p-nNOS Ser847 expression (**f**) (n = 6 per group. *P<0.05 vs. SHAM and TAC+E. ^†^P<0.05 vs. SHAM).

We evaluated the protein expression of eNOS and nNOS isoforms after TAC as well as the role of these proteins in the cardioprotective effects of aerobic exercise. First, we examined the expression and phosphorylation of myocardial eNOS, which is generally phosphorylated at Ser1177 and Ser114. As shown in Fig 7, the total eNOS levels was not changed in any group, whereas increased phospho-eNOS^Ser1177^ levels and decreased phospho-eNOS^Ser114^ levels were observed in the TAC group compared with the SHAM group (P<0.05). Decreased phospho-eNOS^Ser1177^ and increased phospho-eNOS^Ser114^ levels were observed in the TAC+E group compared with the TAC group (P<0.05, Fig 7c and 7d).

Total nNOS protein expression was increased in the TAC group compared with the SHAM group (P<0.05). Meanwhile, exercise training significantly increased total nNOS expression (P<0.05, Fig 7b). Decreased phospho-nNOS^Ser1412^ levels and increased phospho-nNOS^Ser847^ levels were observed in the TAC group compared with the SHAM group (P<0.05, Fig 7e and 7f). Exercise training increased phospho-nNOS^Ser1412^ and decreased phospho-nNOS^Ser847^ expression in the TAC+E group compared with the TAC group (P<0.05, Fig 7e and 7f).

### Cardioprotective effects of aerobic exercise was abolished by β3-AR antagonism

**Fig 8:**
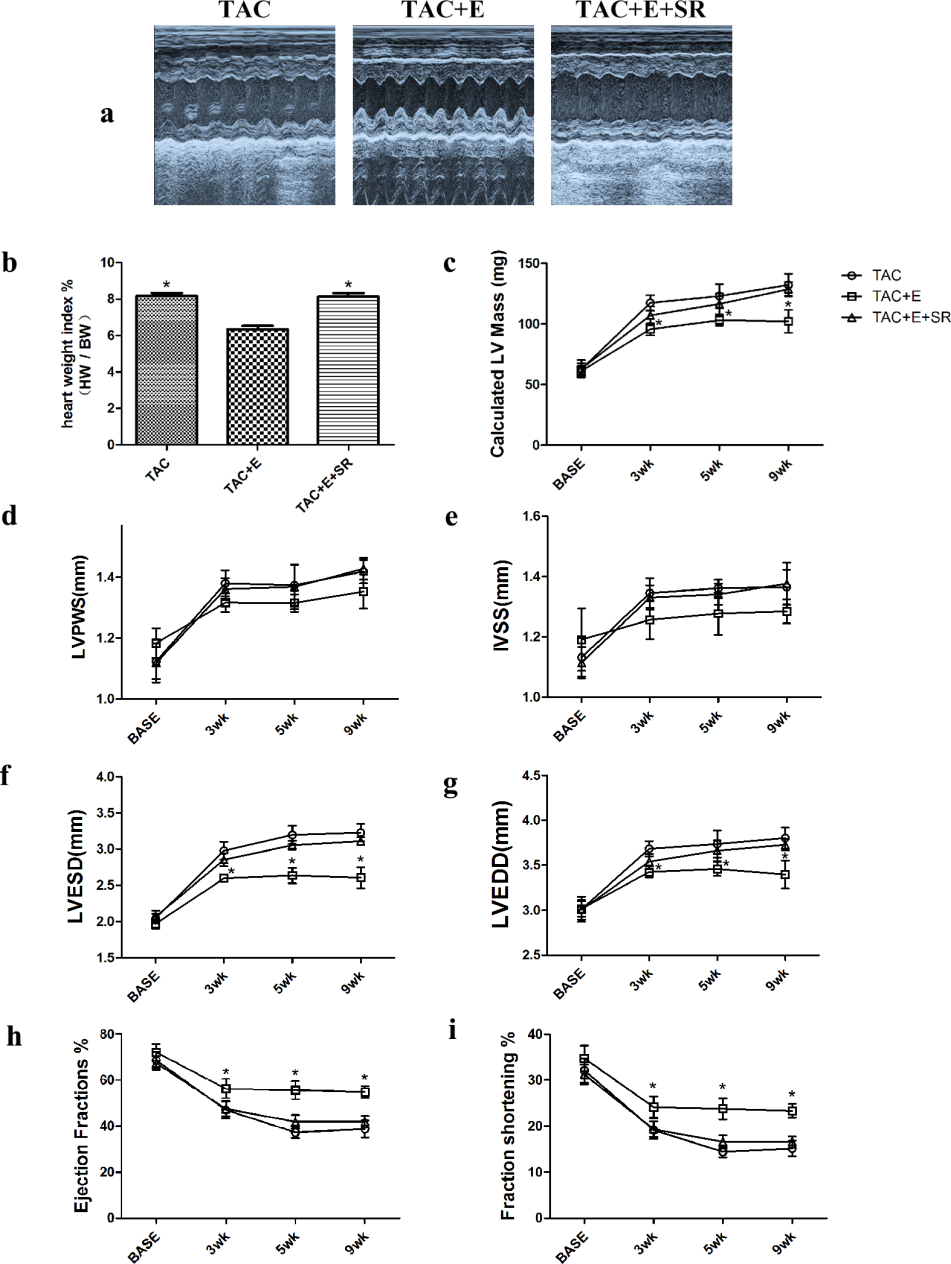
Cardioprotective effects of aerobic exercise was abolished by β3-AR antagonism. (**a**) Representative M-mode echocardiographic images were taken at the level of the papillary muscle, where left ventricular diameters can be measured. (**b**) Representative heart weight to body weight ratio. (n = 12 per group. *P<0.05 vs. TAC+E). (**c**) Quantification of calculated left ventricular mass (LVM). Quantification of calculated left ventricular end systolic posterior wall (LVPWS) (**d**), interventricular septum end systolic thickness (IVSS) (**e**). (**c**) (**d**) (**e**) (n = 12 per group. *P<0.05 vs. TAC and TAC+E+SR). Quantification of the left ventricular end systolic diameter (LVESd) (**f**), left ventricular end diastolic diameter (LVEDd) (**g**), left ventricular ejection fraction (EF) (**h**) and fractional shortening (FS) (**i**). (**f**) (**g**) (**h**) (**i**) (n = 12 per group. *P<0.05 vs. TAC and TAC+E+SR).

To illuminate the mechanism of the cardioprotective effects of aerobic exercise, exercise-trained TAC mice were treated with SR59230A. SR59230A abolished the effect of exercise training in cardiac hypertrophy (Fig 8). The heart weight to body weight ratio in the TAC+E+SR group increased compared with that in the TAC+E group, which were consistent with the LVM (P<0.05, Fig 8b and 8c). Body weight did not differ significantly among the groups at baseline, 3w, 5w and 9w (S1 Fig). IVS and LVPW did not significantly differ in each groups (Fig 8d and 8e). After administrated with SR59230A, the decreased LVESd and LVEDd both returned to the levels observed in the TAC group (P<0.05, Fig 8f and 8g), as did the elevated EF% and FS% (P<0.05, Fig 8h and 8i). Additionally, echocardiographic analysis revealed that the baseline parameters were similar in all groups.

### Effects of β3-AR antagonism on myocyte hypertrophy, fibrosis and catecholamine levels

**Fig 9:**
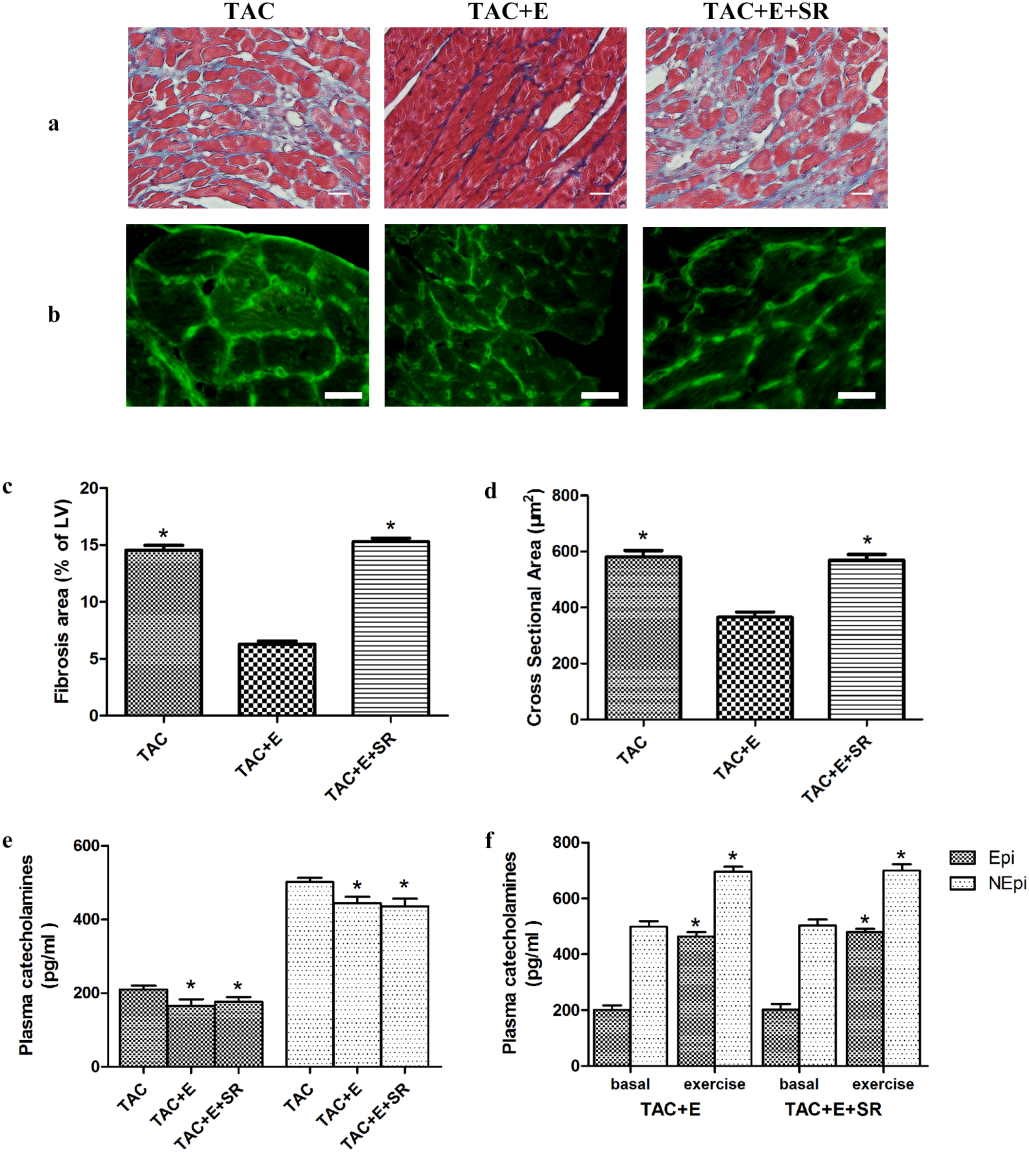
Effects of β3-AR antagonism on myocyte hypertrophy, fibrosis and catecholamine levels. (**a**) Representative Masson’s trichrome staining revealed left ventricular fibrosis 9 weeks after exercise training. Red indicates viable myocardium; blue indicates fibrosis. Scale bar represents 20 μm. (**b**) Representative WGA staining revealed cardiomyocyte cross sectional area. Green fluorescence delineate cardiomyocyte membranes. Scale bar represents 20 μm. (**c**) Quantitative analysis of the fibrotic area (n = 20 per group. *P<0.05 vs. TAC+E). (**d**) Quantitative analysis of cardiomyocyte cross sectional area (n = 100 per group. *P<0.05 TAC+E). (**e**) Quantitative of the plasma catecholamine levels at basal. (n = 5 per group. *P<0.05 vs. TAC) (**f**) Quantification analysis of the plasma catecholamine levels right after exercise. (n = 5 per group. *P<0.05 vs. basal).

Treatment with SR59230A increased the fibrotic area in the TAC+E+SR group compared with that in the TAC+E group (P<0.05, Fig 9c). SR59230A also abolished the effect of aerobic exercise on myocyte hypertrophy. The cardiomyocyte cross sectional area was increased in the TAC+E+SR group compared with TAC+E group (568.70±94.44 μm^2^ vs. 365.92±78.21 μm^2^ in the TAC+E group, P<0.05, Fig 9d). The basal plasma levels of NEpi and Epi were decreased in the TAC+E and TAC+E+SR groups (vs. TAC group, P<0.05, Fig 9e). The plasma levels of both NEpi and Epi were significantly increased right after exercise (P<0.05, Fig 9f).

### β3-AR antagonism abolished the NO production increase and oxidative stress decrease induced by aerobic exercise

**Fig 10:**
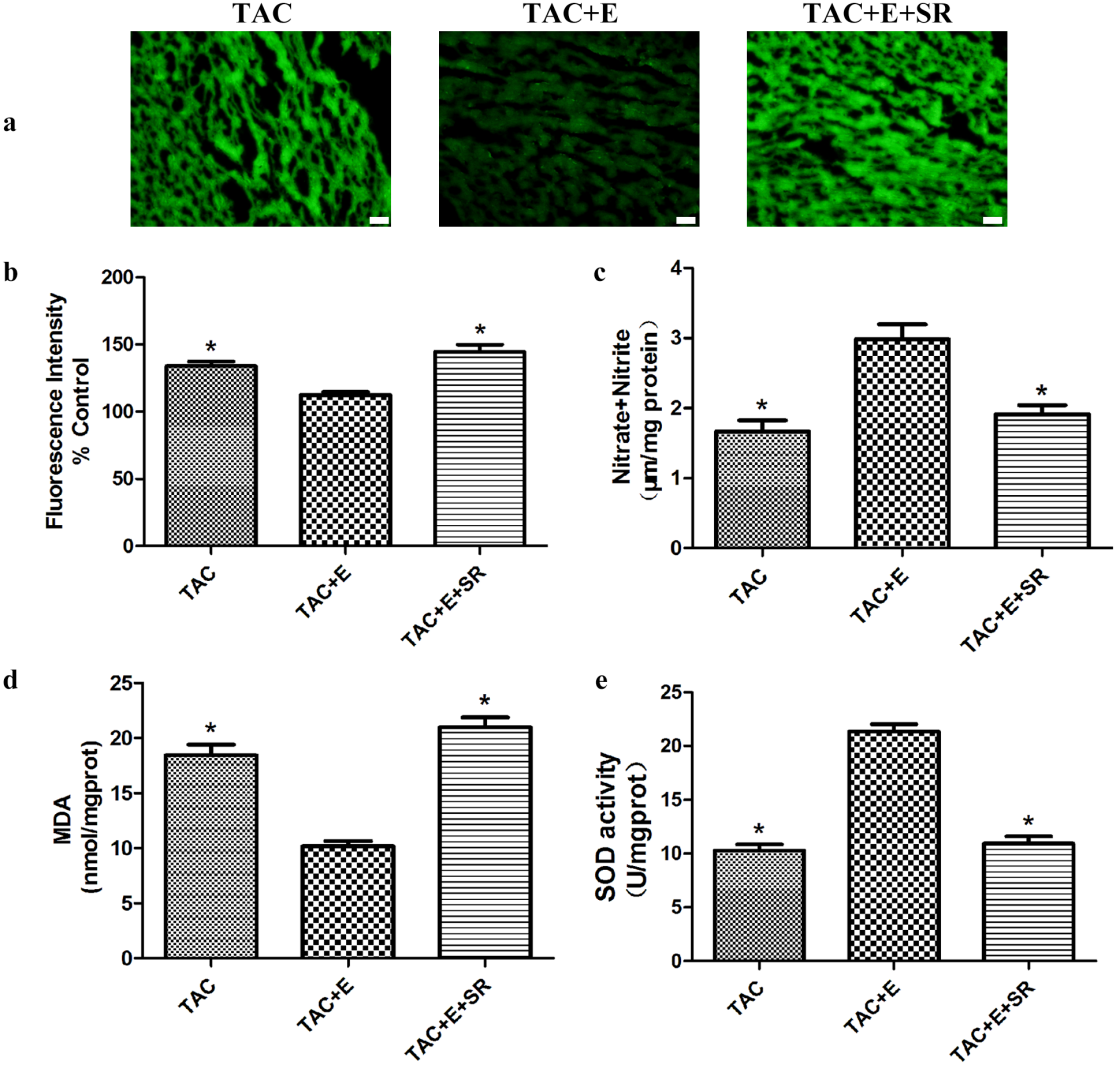
Effects of SR59230A on cardiac NO production and oxidative stress after aerobic exercise. (**a**) Representative images of heart sections fluorescently stained for ROS from the TAC, TAC+E and TAC+E+SR groups. Green fluorescence indicates ROS production. Scale bar represents 50 μm. (**b**) Quantitative analysis of the ROS levels (n = 10 per group. *P<0.05 vs. TAC+E). (**c**) Quantitative analysis of NO production assessed using the nitrate reductase method (n = 8 per group. *P<0.05 vs. TAC+E). (**d**) Quantitative analysis of MDA levels assessed using the TBA method (n = 8 per group. *P<0.05 vs. TAC+E). (**e**) Quantitative analysis of SOD levels assessed using the hydroxylamine method (n = 8 per group. *P<0.05 vs. TAC+E).

Compared with TAC+E group, SR59230A inhibited NO production (1.92±0.22 μm/mg prot, P<0.05, Fig 10c) and increased either the ROS generation (144.54±12.09%, P<0.05, Fig 10b) or the MDA level (20.99±1.55 nmol/mg prot, P<0.05, Fig 10d) in the myocardium. Furthermore, the SOD level was significantly decreased in the TAC+E+SR group (10.93±1.17 U/mg prot vs 21.37±1.14 U/mg prot in the TAC+E group, P<0.05, Fig 10e).

### Aerobic exercise induced β3-AR signaling activation was abolished by SR59230A

**Fig 11:**
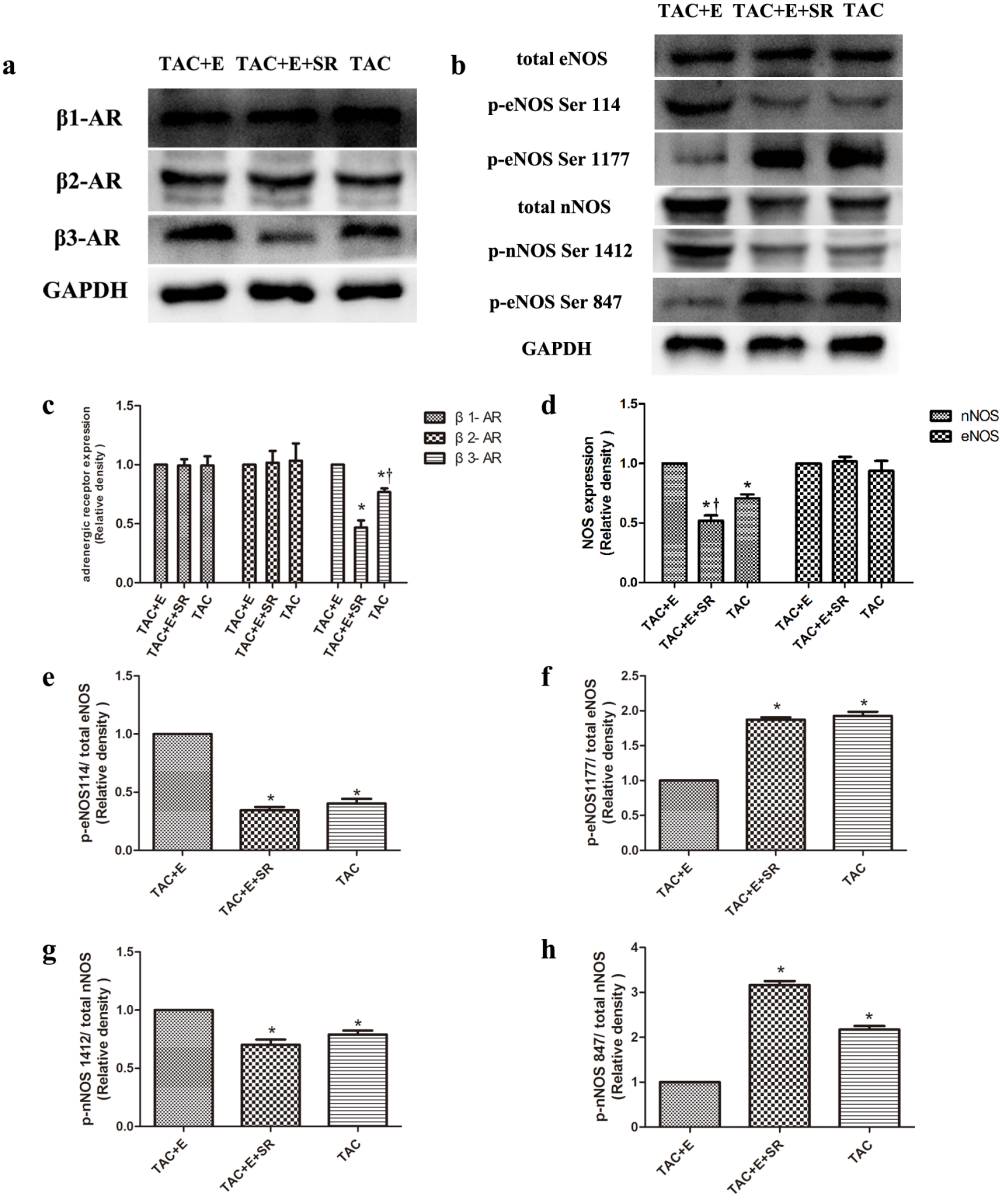
Aerobic exercise induced β3-AR signaling activation was abolished by SR59230A. (**a**) Representative immunoblots of β1-AR, β2-AR and β3-AR in the TAC, TAC+E+SR and TAC+E groups. (**b**) Representative immunoblots of p-eNOS (Ser1177 /Ser114), total eNOS, p-nNOS (Ser1412/Ser847) and total nNOS levels in the TAC, TAC+E+SR and TAC+E groups. (**c**) Semiquantitative analysis of β1-AR, β2-AR and β3-AR expression (n = 6 per group. *P<0.05 vs. TAC+E. ^†^P<0.05 vs. TAC+E+SR). (**d**)Semiquantitative analysis of eNOS and nNOS expression (n = 6 per group. *P<0.05 vs. TAC+E. ^†^P<0.05 vs. TAC). Semiquantitative analysis of p-eNOS Ser114(**e**), p-eNOS Ser1177 (**f**), p-nNOS Ser1412 (**g**) and p-nNOS Ser847 (**h**) (n = 6 per group. *P<0.05 vs. TAC+E).

Cardiac β3-AR expression was significantly decreased in the TAC+E+SR group compared with the TAC+E and TAC groups (P<0.05, Fig 11c). Neither β1-AR nor β2-AR expression was changed in any group (Fig 11c). The total eNOS levels was not changed in any group(Fig 11d), SR59230A significantly increased phospho-eNOS^Ser1177^ levels and decreased phospho-eNOS^Ser114^ levels in the TAC+E+SR group compared with the TAC+E group (P<0.05, Fig 11e and 11f). In addition, SR59230A decreased the expression of both total nNOS and phospho-nNOS^Ser1412^ and increased the expression of phospho-nNOS^Ser847^ in the TAC+E+SR group compared with the TAC+E group (P<0.05, Fig 11d, 11g and 11h).

## Discussion

It is generally accepted that moderate exercise has beneficial effects on the cardiovascular system [21,22]. Previous studies have proposed that cardiovascular diseases such as HF and hypertension are often associated with SNS overactivity [23–25]. Conversely, moderate exercise could reduce SNS overactivity in hypertension and HF [26]. These results are consistent with the present study. In the present study, we observed that 3 weeks of aerobic exercise training partly prevented cardiac dysfunction and the deterioration of LV chamber dilation and partially inhibited pressure overload-induced myocardial hypertrophy. However, the mechanisms by which aerobic exercise exerts this cardioprotective effect remain to be fully elucidated. Earlier studies suggested that catalase and heat shock proteins (HSPs) contributed to the cardioprotective effects of exercise. However, the protective effects were sustained for 9 days following exercise, at which point the increases in catalase and HSP expression had waned, suggesting that some other cardioprotective mechanisms were also involved in this sustained protection [5,27]. Accumulating studies support the finding that sustained insults, such as hypertension and hemodynamic overload, lead to dysfunctional NOS activity and NO production [28–30]. Meanwhile, in a NO-deficient hypertension model, NOS expression in the heart was elevated during exercise [7]. Based on this evidence, NOS/NO were suggested to be involved in the cardioprotective effects of exercise training. An additional question remaining to be answered is what mechanisms lead to NOS activation during exercise. As suggested by previous studies, β-AR is a target for the treatment of cardiovascular diseases [31], and β3-AR is associated with NO release via NOS [32].

Three β-AR subtypes play essential roles in modulating cardiac function. The effects of β1/2-ARs, including positive chronotropic and inotropic effects, have been demonstrated previously. Previous studies suggest that persistent stimulation of β1AR and β2-AR under pathological circumstances such as HF lead to cardiomyocyte apoptosis, cardiomyocyte hypertrophy and maladaptive cardiac remodeling[33–35]. Moreover, persistent stimulation also lead to β1/2-ARs down-regulation or desensitization during HF[11]. Some studies suggested that the expression of β1/2-ARs decreased in failing heart[36–38]. However, other studies have suggested that the expression of β1/2-ARs in the left ventricular myocardium remains unchanged during heart failure[17,39,40]. This discrepancy could be explained by the different species of subjects. The subjects of the β1/2-ARs decreased studies just mentioned were rat or human. However, in C57BL6/J mouse studies, including the present study, β1-AR and β2-AR were unchanged in failing heart as just mentioned. Despite the low levels of β3-AR expression under physiological conditions, accumulating evidence suggests that β3-AR expression is increased and that β3-ARs play a negative inotropic effect in HF [12,41]. Moreover, previous studies suggested that β3-ARs are activated at higher concentrations of catecholamines than are β1/2-ARs [9]. In the present study, we observed that the basal plasma catecholamine levels are increased after TAC, and the levels of crculating catecholamine are significantly increased right after aerobic exercise. Nine weeks of aerobic exercise resulted in a reduction of basal plasma catecholamine levels. However, the basal catecholamine levels of exercise trained TAC mice are still higher than SHAM mice, indicating that β3-ARs could be actived in response to aerobic exercise. Moreover, we observed that the expression of β3-AR was increased in TAC mice after 9 weeks of aerobic exercise. Consistent with our results, previous study also reported that the protein expression of β3-AR increased in myocardial infarction rats after 8 weeks of aerobic exercise[42]. However, the relationship between the plasma catecholamine levels and the protein expression of β3-AR is still unknown.

β3-ARs plays a vital protective role under conditions of sympathetic overstimulation [32]. Our previous study demonstrated that β3-AR-specific agonism (BRL37344) preserved heart functional recovery after pressure overload-induced hypertrophy and cardiac systolic dysfunction [15]. Similarly, a previous study demonstrated that the β1-blocker nebivolol, another selective β3-AR agonist, could reduce the cardiac infarct size in mice subjected to myocardial ischemia and reperfusion injury [43]. Moreover, our previous results showed that mice lacking β3-AR (β3^−/−^) had greater LV dilation, myocyte hypertrophy, worse systolic function and enhanced fibrosis after TAC [8]. In the present study, we found that the cardioprotective effects of aerobic exercise training partly occurred through β3-AR stimulation in failing hearts. To verify this finding, the β3-AR-specific antagonist SR59230A was used. Notably, the cardioprotective effect of exercise could be abolished by treatment with SR59230A. In previous studies, the dose of SR59230A used in the present study effectively inhibited β3-AR, and caused no reported myocardial toxicity or other side effects [17,39]. Taken together, these results indicate that the cardioprotective effects of aerobic exercise training are closely related to β3-AR stimulation.

Many pieces of evidence have revealed that sustained stressors lead to NOS/NO dysfunction and ROS activation in the cardiovascular system [44]. Our results revealed decreased NO production and increased ROS production in hypertrophic and failing heart. Consistent with our findings, Calvert et al. [45] suggested that the NO metabolites were increased during exercise. In our previous study, we observed a reduction of TAC-induced superoxide generation by BRL treatment [15]. In present study, we found that TAC-induced ROS generation was inhibited by aerobic exercise training and that this inhibition was abolished by treatment with SR59230A. These findings are in line with those of our previous study, that the cardioprotective effects of β3-AR stimulation on cardiac hypertrophy and HF can be attributed to the equilibrium of NO and ROS production [15].

MDA and SOD levels can indirectly reflect free radical metabolism and oxidative stress. MDA is the product of lipid peroxidation by poly-unsaturated fatty acids and oxygen free radicals, and the MDA content may reflect the severity of membrane damage, which could indicate the levels of free radicals and oxidative stress[46]. The relationship between MDA and exercise remains controversial. Z.N.O. Kumral et al. [47] found that in rats with renovascular hypertension (RVH)-induced cardiac dysfunction, the cardiac MDA level was significantly increased in sedentary RVH rats and that exercise training performed after the onset of RVH abolished the increase and returned cardiac MDA to control levels. Balci and Pepe [48] also reported that cardiac MDA levels in rats was decreased after endurance exercise training, while others have observed increased cardiac MDA levels induced by exhaustive swimming exercise in rats [49,50]. The controversial findings could be explained by the different types of exercise used in those studies. In the present study, we found that MDA production was suppressed by aerobic exercise training after TAC. SOD, an important antioxidant enzyme in vivo, plays a crucial role in maintaining the balance between oxidation and anti-oxidation and could protect cells against oxidative stress [46]. SOD levels may reflect the ability to scavenge free radicals. In accordance with previous study [51], our results suggest that SOD activity was increased by aerobic exercise training. Furthermore, the decrease in MDA and the increase in SOD induced by aerobic exercise was abolished by treatment with SR59230A. Taken together, these results indicate that the cardioprotective effects of aerobic exercise training could be attributed to the suppression of oxidative stress via β3-AR stimulation.

Three NOS isoforms (eNOS, nNOS and iNOS) are involved in NO release, but which one is involved in the regulation of myocardial function remains unknown. Previous studies have suggested a role for eNOS/NO in exercise-mediated cardioprotection [52]. A recent study suggested that the beneficial cardiac adaptations observed after exercise training were mediated via enhanced nNOS signaling [3]. Some studies also demonstrated that β3-AR modulates NO signaling through nNOS or iNOS [45]. Our previous study suggested that nNOS as the primary downstream NOS isoform of β3-AR in maintaining NO in HF [15]. Meanwhile, we found that eNOS and nNOS may be associated with the cardioprotective effects of β3-AR against injury due to MI [17]. Therefore, we detected the expression and activation of eNOS and nNOS in the present study.

eNOS activity is generally modulated by either translocation or phosphorylation. However, the translocation of eNOS was only observed in the right atrium, not in the left ventricle[53]. In the present study, we found that total eNOS protein expression was unchanged by aerobic exercise training, whereas eNOS phosphorylation at Ser1177 was decreased, and eNOS phosphorylation at Ser114 was increased. Since it has been reported that Ser1177 phosphorylation activates eNOS, while Ser114 phosphorylation deactivates eNOS [54,55], the present data suggest that β3-AR stimulation led to the deactivation of eNOS in the failing myocardium after aerobic exercise training, which is consistent with our previous results [15]. Napp et al. [14] also suggested that eNOS was deactivated by β3-AR stimulation in failing myocardium isolated from human. NO production induced by β3-AR stimulation exhibited a negative inotropic effect, while eNOS was deactivated in failing myocardium. This contradiction could be explained by the activation of other NOS isoforms. The present study revealed that nNOS protein levels were increased and that nNOS was activated by aerobic exercise training-mediated β3-AR stimulation. Previous studies suggested that Ser1412 phosphorylation activates nNOS, and enhancing NO production, while Ser847 phosphorylation inactivates nNOS, suppressing NO production [56,57]. In the present study, Ser1412 phosphorylation increased, and Ser847 phosphorylation decreased after aerobic exercise training, indicating nNOS activation. Meanwhile, aerobic exercise training mediated NO production was also increased. Moreover, increased nNOS protein expression and activation associated with increased NO production induced by aerobic exercise training can be abolished by treatment with the β3-AR-specific antagonist SR59230A. These results further suggest that nNOS-derived NO production is the primary source of the cardioprotective effect of aerobic exercise training via β3-AR stimulation in failing myocardium.

In conclusion, the present study provides evidence that moderate aerobic exercise training in mice could improve systolic function and recover pathological remodeling in failing hearts as well as alleviate cardiac fibrosis and hypertrophy, and these effects are closely related to β3-AR activation. In addition, nNOS-mediated NO production and reduced oxidative stress may be associated with the protective effect of β3-ARs. These data indicate a mechanism by which the β3-AR-nNOS-NO pathways are related to the protective effect of aerobic exercise training.

## Supporting information

S1 Fig Body weight of mice did not differ significantly among groups. (**a**) Quantitative analysis of the body weight in SHAM, SHAM+E, TAC and TAC+E groups. (**b**) Quantitative analysis of the body weight in TAC, TAC+E and TAC+E+SR groups. (**a**) (**b**) (n = 12 per group).
(TIF)
