# Supplementary figures and images for: Aerobic exercise protects against pressure overload-induced cardiac dysfunction and hypertrophy via β3-AR-nNOS-NO activation
Source: PLoS One. 2017 Jun 16;12(6):e0179648. doi: 10.1371/journal.pone.0179648 (PMC5473571; doi:10.1371/journal.pone.0179648)

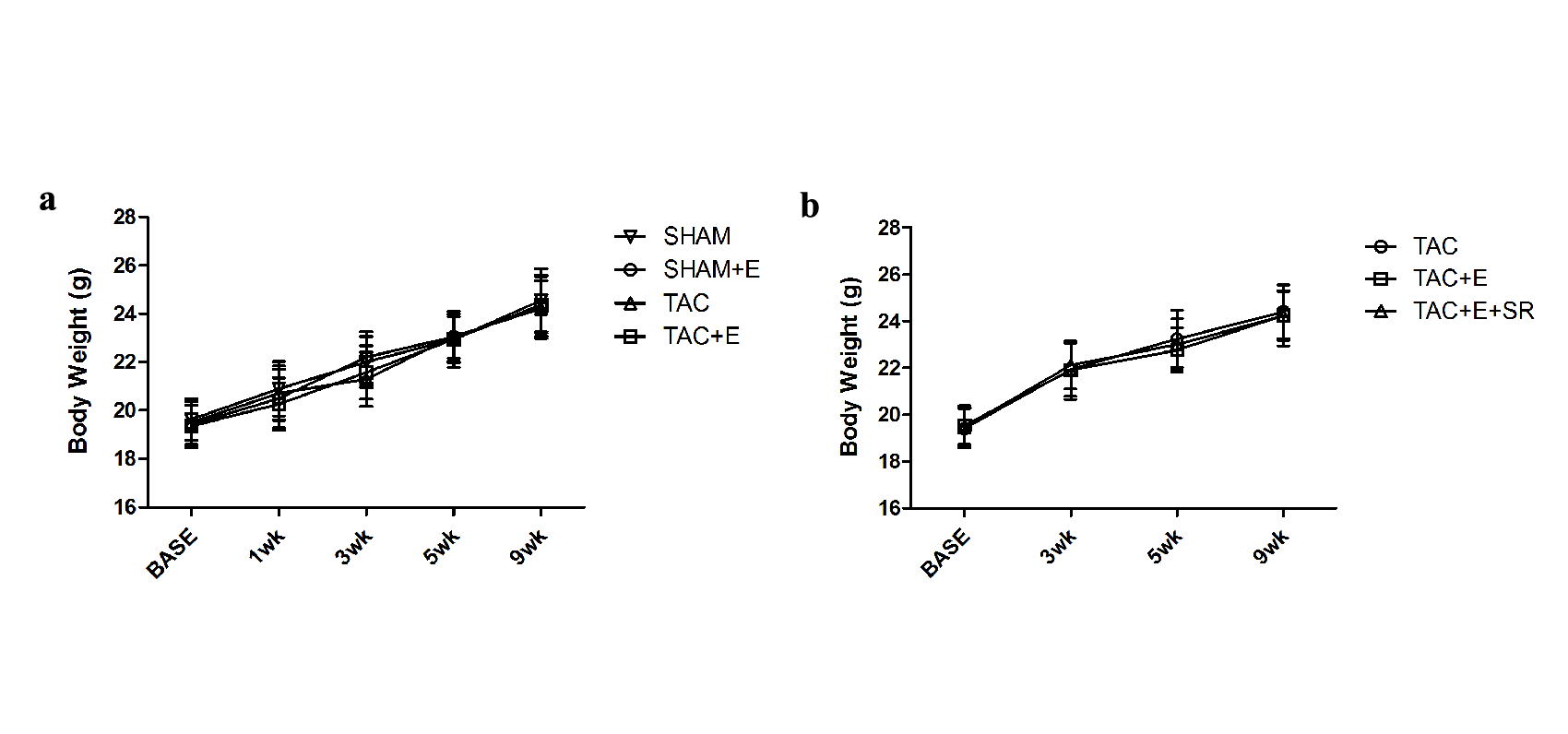

Supplement: S1 Fig — (a) Quantitative analysis of the body weight in SHAM, SHAM+E, TAC and TAC+E groups. (b) Quantitative analysis of the body weight in TAC, TAC+E and TAC+E+SR groups. (a) (b) (n = 12 per group). (TIF) [file pone.0179648.s001.tif]
